# Psychrophiles elude genomic prediction of optimal growth temperature

**DOI:** 10.1128/msystems.00368-26

**Published:** 2026-07-21

**Authors:** Alexandra Walling, Michael Hoffert, Noah Fierer, J. L. Weissman

**Affiliations:** 1 Institute for Advanced Computational Science, Stony Brook University 12301, Stony Brook, New York, USA; 2 Department of Ecology & Evolution, Stony Brook University 171425, Stony Brook, New York, USA; 3 Cooperative Institute for Research in Environmental Sciences, University of Colorado 1877 , Boulder, Colorado, USA; 4 Department of Ecology and Evolutionary Biology, University of Colorado 118570, Boulder, Colorado, USA; CNRS Delegation Bretagne et Pays de Loire, Nantes, France

**Keywords:** optimal growth temperature, traits, temperature, microbial growth

## Abstract

While there have been numerous efforts to use genomic information to predict the optimal growth temperatures of bacteria and archaea, such models are often highly inaccurate when applied to taxa with lower optimal growth temperatures, including “cold-loving” psychrophiles. Toki et al. explain why this might be the case, namely, that adaptations to low-temperature growth evolved more recently and are thus less phylogenetically conserved than adaptations to growth at higher temperatures. Leveraging this information about evolutionary history, S. Toki, M. Matsui, K. Tominaga, T. K. Suzuki, et al. (mSystems 11:e00062-26, 2026, https://doi.org/10.1128/msystems.00062-26) offer a solution that uses gene presence/absence information instead of models based solely on amino acid composition to predict the optimal growth temperatures of psychrophiles.

## COMMENTARY

Most prokaryotic taxa remain difficult to grow and study in culture, and their environmental preferences remain largely unquantified (1). As one example, we know that the optimal temperature for growth (OGT) can vary dramatically across prokaryotes, but the OGT values for most prokaryotic species found on Earth remain unknown. As OGT is an important determinant of when and where species will grow, being able to reliably infer OGT values is useful for many reasons, from identifying effective culturing strategies to predicting how the distributions of microbial taxa will shift in response to climate change (1). Because the phylogenetic coherence of this trait is weak, with even members of the same genus having variable OGTs (2), we cannot simply predict OGT values from taxonomic or phylogenetic information alone (3). Instead, given the vast quantity of genomic information now available for uncultured prokaryotes, there is broad interest in developing genome-based models to infer OGT for uncultured species. Pre-existing modeling efforts have leveraged various genomic features to infer OGT values with varying degrees of success. Examples of such models include those based on nucleotide sequences (4–10); amino acid sequences and protein annotations (8, 11–14); and a combination of features derived from DNA and protein data (15). Yet, because trait axes orthogonal to OGT also impact genome composition, composition alone is not sufficient to explain OGT differences between thermophiles and psychrophiles (16, 17).

**Fig 1:**
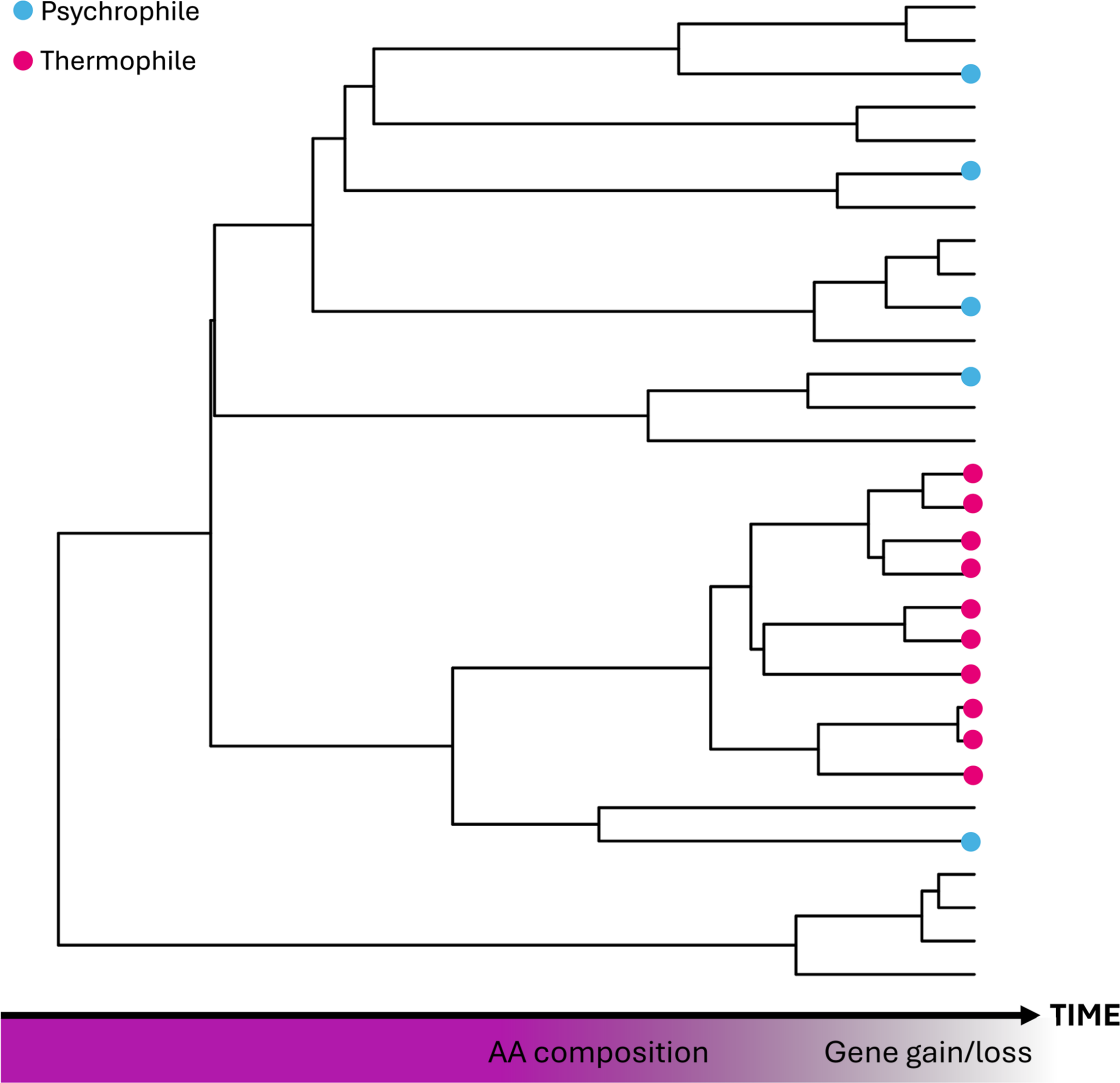
Conceptual figure showing the timescales on which thermophily and psychrophily evolve, and thus what kinds of genomic traits are appropriate to serve as predictors of thermophily and psychrophily.

Despite extensive efforts, developing accurate genome-based models of OGT has proven to be a persistent challenge. Training data sets for OGTs from cultured isolates are limited, both because of the narrow temperature ranges at which most isolates grow (25°C to 35°C), discrete annotation in many databases at common incubator temperatures (for instance, 25°C, 30°C, and 37°C), or isolates binned into broad categories (e.g., thermophile versus mesophile) rather than measuring OGT as a continuous variable (15, 18, 19). Culture biases mean that few annotated strains are available with low observed OGT (in line with slow-growing bacteria often lacking cultured representatives [20]). Moreover, observed environmental temperatures provide only limited information about temperature preferences. Previous work on experimentally derived minimum and optimum growth temperatures for Antarctic soil and marine bacteria has found that OGTs are much higher than we might expect from the corresponding environmental temperatures, in the range of typical mesophiles, having perhaps evolved as a buffer against death from heat stress (21, 22).

Thus, while genomic models are often effective at differentiating mesophiles from thermophiles, they frequently fail to predict OGT differences among mesophiles and psychrophiles. Kurokawa et al. (23), for instance, struggled to predict OGT for psychrophiles in stream and ocean samples from amino acid fractions alone. To tackle this problem, Toki et al. (24) developed a new model for OGT prediction by surveying genomic features near start codons, under the hypothesis that the signals of adaptation to lower temperature growth would be more evident closer to the start codon due to inhibition of translation initiation by mRNA secondary structures. In total, they incorporated 526 genomic features into model training, including tRNA, rRNA, amino acid, and codon base composition, GC content, minimum free energy of tRNA and rRNA, and, to improve the prediction of psychrophiles, features around the start codon. Given that genome-based trait models trained on certain taxa often fail when applied to lineages underrepresented in the training data (1, 2, 15), they tested model accuracy with “leave-one-out” cross-validation across phyla. This initial OGT model improved overall accuracy, but OGT prediction for psychrophiles remained difficult, with higher mean average error compared to other temperature ranges.

Leveraging phylogenetic information, they found that thermophiles cluster in closely related clades while psychrophiles are phylogenetically dispersed and have more recently adapted to low temperatures. Ancestral state reconstruction revealed that psychrophilic lineages typically experienced recent decreases in OGT (Fig. 1). Because genome-wide amino acid composition, a common predictor in genomic OGT models (9, 10, 23), evolves slowly, psychrophiles may not have a characteristic amino acid profile as is evident in lineages with higher OGTs. Instead, Toki et al. (24) used gene presence/absence in place of amino acid composition to improve psychrophile OGT predictions, including OGT-associated genes conserved across many bacterial lineages and OGT ranges. Incorporating gene copy number information into their model for OGT notably improved prediction of OGT of psychrophiles, particularly for species with OGTs below 5°C.

There is still room to improve the accuracy of genome-based models of prokaryotic OGTs. First, we need high-quality OGT data across a broad diversity of prokaryotes for model validation. These data on OGTs, as well as other relevant traits, would ideally come from high-throughput assays where traits are experimentally quantified *in vitro* using consistent methods. Second, model feature space can be expanded; Toki et al. (24) suggest that single-nucleotide substitutions as well as gene presence/absence can contribute to the evolution of OGT. Both nucleotide substitutions and gene loss/acquisition reflect the complex influence of temperature on the structure and performance of biomolecules and processes. AI-enabled protein structural prediction also provides an exciting pathway forward for continued model development (25, 26). However, model improvements must be paired with the development of trait databases via high-throughput experimentation to address the lack of high-quality trait data, a major hurdle for genome-based microbial trait prediction.

Overall, Toki et al. provide insights into the evolutionary mechanisms producing varying patterns in temperature preferences across prokaryotes, insights that are broadly relevant to ongoing efforts to build genome-based models to infer traits and environmental preferences for the majority of taxa that remain uncultivated.
